#  Role of Human Polyomaviruses in Respiratory Tract Disease in Young Children

**DOI:** 10.3201/eid1411.080394

**Published:** 2008-11

**Authors:** Rachel L. Wattier, Marietta Vázquez, Carla Weibel, Eugene D. Shapiro, David Ferguson, Marie L. Landry, Jeffrey S. Kahn

**Affiliations:** Yale University School of Medicine, New Haven, Connecticut, USA

**Keywords:** Human polyomavirus, WU, KI, clinical features, respiratory infections, dispatch

## Abstract

KI virus was detected in respiratory secretions of 8/367 (2.2%) symptomatic and 0/96 asymptomatic children (p = 0.215). WU virus was detected in 26/367 (7.1%) symptomatic and 6/96 (6.3%) asymptomatic children (p = 1.00). These human polyomaviruses may not independently cause respiratory tract disease in young children.

In 2007, 2 new human polyomaviruses, KI virus (KIV) and WU virus (WUV), were identified by molecular screening of respiratory secretions from children <2 years of age with symptomatic respiratory tract disease (*1*,*2*). Both viruses have since been detected in asymptomatic children and in those concurrently infected with other respiratory viruses, suggesting that KIV and WUV may not cause respiratory tract disease (*3*–*5*). To further understand the epidemiology of these viruses in young children and to clarify their association with symptomatic respiratory tract infections in this age group, we screened respiratory specimens from both asymptomatic and symptomatic children for the presence of KIV and WUV.

## The Study

Respiratory specimens from 2 groups of children (all <2 years of age) were collected in 2004 and screened for KIV and WUV. The first group comprised symptomatic children whose respiratory specimens were submitted to the Clinical Virology Laboratory, Yale–New Haven Hospital, New Haven, Connecticut. These respiratory specimens tested negative for respiratory syncytial virus (RSV), parainfluenza viruses (types 1–3), influenza viruses A and B, and adenovirus by direct fluorescence antibody assay. The second group comprised asymptomatic children at the hospital-affiliated pediatric clinic for well-child care. Nucleic acids were extracted from each specimen by using QIAamp nucleic acid purification kits (QIAGEN, Valencia, CA, USA). Samples were screened by nested PCR for both KIV and WUV (for WUV, the first primers were those used by Gaynor et. al., and the nested primers were 5′-GCGCATCAAGAGGCACAGCTACTATTTC-3′ and 5′-GCGCCTAGCCTGTGAACTCCATC-3′). The G/C clamp for each primer is underlined (*1*,*2*). Positive and negative controls were included in each set of PCRs. All PCR products were sequenced. Any child who had multiple specimens with positive results was included once in the total number of children whose specimens tested positive for a given virus.

Specimens from symptomatic children who tested positive for KIV or WUV were also screened for human bocavirus (HBoV); human metapneumovirus (hMPV); human coronaviruses (HCoV) 229E, NL63, and HKU1; and human picornaviruses (including rhinoviruses [HRV]) by using previously described methods (*6*–*12*). To screen for human parainfluenzavirus type 4 and HCoV OC43, RNA extraction and reverse transcription were performed as previously described (*7*). The primers used to amplify hPIV4 were 5′-GCGAGAGGATCCAGCTGGTGGC-3′ and 5′-GCGCCCTAATCTTTCCTGTTGATGG-3′. The primers for HCoV-OC43 were 5′-GCATAAGCCCCGCCAGAAGAGGAG-3′ and 5′-GCGCTGACGCTGTGGTTTTGGACT-3′.

We tested 423 direct fluorescent antibody–negative respiratory specimens, from 367 children, for KIV and WUV. The results of screening are summarized in the Table. Of the 367 symptomatic children, there were 8 (2.2%; 95% confidence interval [CI] 1.0%–4.3%) whose specimens tested positive for KIV and 26 (7.1%; 95% CI 4.7%–10.2%) whose specimens tested positive for WUV. One child had 2 specimens that tested positive for WUV. None (0%; 95% CI 0%–4.0%) of the 96 specimens from asymptomatic children tested positive for KIV. Specimens from six (6.3%; 95% CI 2.3%–13.1%) of the 96 asymptomatic children tested positive for WUV. The odds ratio for the proportions of symptomatic and asymptomatic children positive for WUV was 1.14 (95% CI 0.46–2.86; p = 1.0, Fisher exact test). The odds ratio for the proportions of symptomatic and asymptomatic children positive for KIV is undefined (none of the specimens from children in the asymptomatic group tested positive). The difference was not statistically significant (p = 0.215). The distribution of number of samples screened per month was similar for the asymptomatic and symptomatic groups.

**Table:** Detection of KIV and WUV in children <2 years of age with and without respiratory tract disease*

Patient group	No. KIV-positive children/total no. tested (%*)*	No. WUV-positive children/total no. tested (%*)*
Respiratory tract disease	8/367 (2.2)†	26/367 (7.1)‡
Asymptomatic	0/96 (0)	6/96 (6.3)

*KIV, KI virus; WUV, WU virus. †p = 0.215, compared with asymptomatic children. ‡p = 1.0, compared with asymptomatic children.

**Figure 1:**
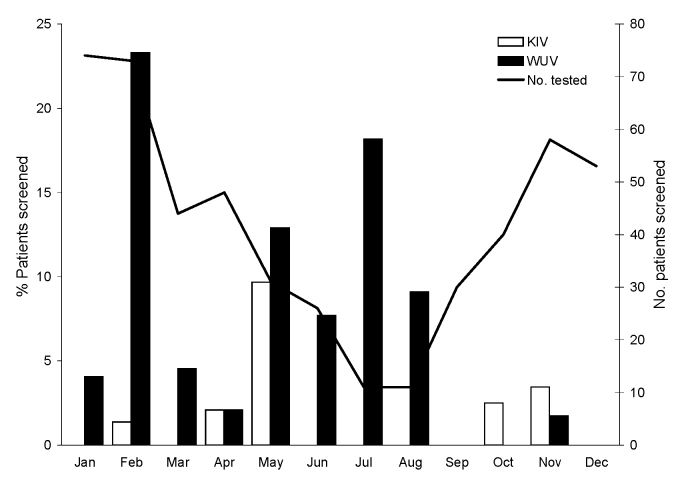
Monthly distribution of children positive for KI virus (KIV) and WU virus (WUV). The WUV-positive children include both asymptomatic and symptomatic children whose specimens tested positive for WUV. One child who tested positive for WUV in February and March is represented in both months. The superimposed line graph represents the number of children tested in each month.

**Figure 2:**
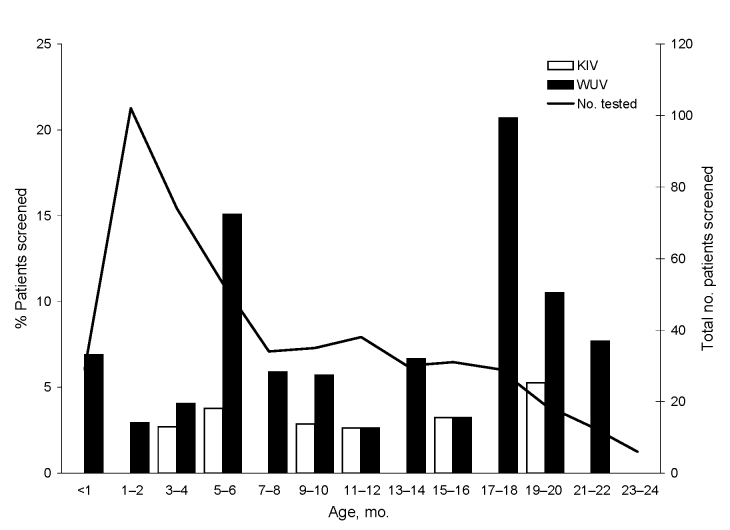
Age distribution of children positive for KI virus (KIV) and WU virus (WUV). The WUV-positive children include both asymptomatic and symptomatic children. One child whose specimens tested positive at age 6 months and again at age 9 months is represented in both age groups. The superimposed line graph represents the number of children tested in each age group.

The monthly distribution of children positive for KIV or WUV is shown in Figure 1. The age distribution of KIV- and WUV-positive children is shown in Figure 2. The youngest KIV-positive child was 4 months of age. The youngest WUV-positive child was 12 days of age.

Viral co-infection was detected in 2 (25%) of the 8 KIV-positive specimens; 1 specimen tested positive for both HBoV and hMPV, and the other tested positive for HBoV. Of the 6 KIV-positive children whose specimens tested negative for other respiratory viruses, all had evidence of respiratory tract disease or fever. Five (63%) of the KIV-positive children had underlying illnesses.

Viral co-infection was detected in 13 (50%) of the 26 WUV-positive symptomatic children; 7 (26%) were positive for hMPV, 4 (15%) for HRV, 2 (7%) for HCoV-NL63, and 2 (7%) for HBoV. Two WUV-positive specimens tested positive for both hMPV and HBoV. Of the 13 WUV-positive children whose specimens tested negative for the other respiratory viruses, 12 had records available for review. Nine (69%) had evidence of respiratory tract disease, including fever. Two of these children had potential noninfectious explanations for their symptoms. One female child had end-stage pulmonary hypertension, and it was unclear whether her respiratory distress was due to worsening pulmonary hypertension or an infectious process. Respiratory distress developed in a male child after suspected aspiration; his symptoms resolved within 6 hours.

Three of the WUV-positive children had been hospitalized since birth; specimens from 1 of these children tested positive for WUV on 2 occasions. The interval between the first and second specimen was 98 days, and during that time 3 other specimens from this child tested negative for WUV. The child was hospitalized throughout this period and received mechanical ventilation for chronic lung disease. This child and 2 others were hospitalized in the same respiratory care unit at the time of WUV detection. Another child whose specimens tested positive for WUV had recently been discharged from that respiratory care unit.

Of 25 WUV-positive children whose records were available for review, 11 (44%) had underlying illnesses. Of the 6 asymptomatic children whose specimens tested positive for WUV, 1 had a history of prematurity and the other 5 had no underlying illnesses.

## Conclusions

We detected KIV and WUV in respiratory samples obtained from children in Connecticut in 2004. The rates of detection for the symptomatic children in our study are similar to those observed in prior studies (*1*,*3*,*4*). As in studies by others, we detected WUV in asymptomatic and symptomatic children (*3*–*5*,*13*). This finding provides further evidence that asymptomatic infection of WUV may occur. KIV was not detected in any of the asymptomatic children we tested. However, it is possible that subclinical infection with KIV occurs and that we failed to detect any cases because of the low prevalence of KIV in our study population. Our study had only 15% power to detect a difference between 2.2% and 0% for KIV, whereas it had adequate power (90%) to detect a difference between 7.1% and 0% for WUV.

Co-infection with other respiratory viruses was a common finding in both KIV- and WUV-positive children. Because specimens from symptomatic children were tested for common viruses by antigen testing rather than PCR, which is presumably more sensitive, the co-infection rate could, in fact, be greater. The high rates of co-infection observed in our study and the studies of others support the notion that KIV and WUV may not cause respiratory tract disease. However, KIV or WUV was the only virus detected for a small number of children with evidence of respiratory tract disease, so it is still possible that these viruses contribute to respiratory tract disease in susceptible children.

Detection of WUV in 3 patients hospitalized since birth suggests the potential for nosocomial, congenital, or perinatal infection. One of these children had 2 respiratory specimens positive for WUV. Le et al. recently reported detection of WUV in multiple samples obtained from 2 immunocompromised patients (*13*). This finding may represent persistent or latent infection.

In conclusion, we have established that 2 recently discovered polyomaviruses, KIV and WUV, are circulating in Connecticut. Because of the high rates of viral co-infection and detection of WUV in both asymptomatic and symptomatic children, these viruses may not be respiratory pathogens.
